# Diagnostic Sensitivity of NLR and PLR in Early Diagnosis of Gastric Cancer

**DOI:** 10.1155/2020/9146042

**Published:** 2020-03-07

**Authors:** Tianyi Fang, Yimin Wang, Xin Yin, Zhao Zhai, Yu Zhang, Yongheng Yang, Qi You, Zhiguo Li, Yan Ma, Chunfeng Li, Haibin Song, Huawen Shi, Yongle Zhang, Xuefeng Yu, Hongyu Gao, Yihua Sun, Rui Xie, Yingwei Xue

**Affiliations:** ^1^Department of Gastrointestinal Surgery, Harbin Medical University Cancer Hospital, Harbin Medical University, Harbin, Heilongjiang 150086, China; ^2^Department of Pathology, Harbin Medical University, Harbin, China; ^3^Department of Clinical Laboratory, Harbin Medical University Cancer Hospital, Harbin, Heilongjiang 150086, China; ^4^Department of Digestive Internal Medicine and Photodynamic Therapy Center, Harbin Medical University Cancer Hospital, Harbin Medical University, Harbin, Heilongjiang 150086, China

## Abstract

The neutrophil-lymphocyte ratio (NLR) and the platelet-lymphocyte ratio (PLR) are markers of systemic inflammation. However, there is little evidence of the value of inflammation in the early diagnosis of gastric cancer (GC). A total of 2,606 patients diagnosed with GC in the past three years and 3,219 healthy controls over the same period were included in this study. Peripheral blood samples were obtained to analyze the NLR, PLR, carcinoembryonic antigen (CEA), and carbohydrate antigen 19-9 (CA19-9). The optimal cutoff levels for the NLR and PLR were defined by receiver operating characteristic (ROC) curve analysis (NLR = 2.258, PLR = 147.368). The value of different biomarkers for diagnosing GC was compared by the area under the curve (AUC). The NLR and PLR showed diagnostic sensitivity in GC (AUC = 0.715, AUC = 0.707). Using the Bonferroni correction, the NLR and PLR were superior to CEA and CA19-9 in the diagnosis of GC (*P* < 0.0001). The systemic inflammatory markers were significantly higher in the early stage of GC than tumor markers. After grouping patients and healthy controls by gender, we found that the diagnostic significance of combined NLR and PLR for GC was greater in male patients than in female patients (*P* < 0.0001). The diagnostic value of the NLR and PLR in GC is higher than that of the traditional tumor markers CEA and CA19-9. Systemic markers of inflammation are more valuable in male than female patients.

## 1. Introduction

Gastric cancer (GC) remains an important cause of cancer mortality in East Asia, Eastern Europe, and South America with more than 700,000 deaths annually [1]. More than 70% of new cases and deaths are from developing countries, resulting in a social burden that cannot be ignored [2]. With the improvements in detection technology and increasing awareness of health care in recent years, some patients can receive systemic treatment at an early stage, but the five-year survival rate continues to be lower than 10% for patients with advanced GC in China [3–6]. Therefore, the development of inexpensive, robust, quantitative, and powerful biomarkers to diagnose CG in the early stages is crucial.

Since the concept of circulating tumor cell (CTC) was first proposed in 1896, it has been used to detect cancers [7–9]. Researchers looked to capture tumor cells circulating in the blood to improve the rate of early diagnosis of cancers. However, in most regions, the capture of tumor cells is limited by rarity, heterogeneity, and high cost and cannot be routinely used in cancer patients, especially in high-risk populations [10]. Cancer cells that easily enter the peripheral blood in the early stages, but rarely cause distant metastases, have received widespread attention. Inflammation is a known major driver of tumor development and progression, whether in the blood or in the tumor tissue. Traditional immunohistochemistry has the disadvantages of heterogeneity and inconsistent evaluation methods for the detection of immune infiltration in pathological sections. It is also difficult to detect deep lesions in high-risk populations. The immunohematological reaction is an extremely sensitive defense system, and a small amount of bacteria entering the blood causes a transient increase in intravascular inflammatory cells. Previous studies have reported that cancer cells entering the peripheral blood also cause varying degrees of immune cell activation. Therefore, this immune response may be a reliable research approach for the early diagnosis of GC.

In 2018, *CELL* summarized noncancerous tumor-derived cells in circulation such as cancer-associated macrophage-like cells and cancer-associated fibroblasts which may restrict the activity of CTC, especially in the early stages of cancer [10]. The control of systemic immune responses and immune surveillance depends on the regulated trafficking circulating lymphocytes [11]. In 2013, Herzog et al. [12] first reported that the platelet aggregation is not only essential for hemostasis but also critical for immune responses. The neutrophil-lymphocyte ratio (NLR) and platelet-lymphocyte ratio (PLR) have been considered indicators of systemic inflammation in many current clinical studies, when patients have no obvious infection [13]. Therefore, the evaluation of such inexpensive and readily available prognostic markers is urgently needed for large numbers of samples.

In this study, we conducted a retrospective analysis to evaluate the accuracy of serum NLR and PLR in patients diagnosed with GC and compared them with the traditional tumor markers carcinoembryonic antigen (CEA) and carbohydrate antigen 19-9 (CA19-9). We also elucidated the relationship between the systemic inflammatory biomarkers and clinical phenotypic characteristics of GC.

## 2. Materials and Methods

### 2.1. Patient Characteristics

A total of 2,606 patients with GC in the Department of Gastrointestinal Surgery, Harbin Medical University Cancer Hospital, from January 2015 to July 2018 were randomly selected. The diagnosis was based on a tissue sample obtained during gastroscopy and confirmed by postoperative pathology. During hospitalization, all patients underwent abdominal ultrasonography, stomach computed tomography/magnetic resonance imaging, chest radiography, and electrocardiogram, and some patients underwent positron emission tomography-computed tomography when necessary. Patients were staged according to the American Joint Committee on Cancer (AJCC)/Union for International Cancer Control (UICC) 8th edition staging system. The exclusion criteria for patients were as follows: (a) preoperative radiotherapy and/or chemotherapy; (b) concurrent abdominal, lung, intestinal, and other systemic infections and severe cardiovascular disease; (c) antiplatelet agent therapy within the previous three months; (d) patients with steroid therapy when admitted; (e) recurrent GC; (f) patients with blood malignancies and multiple myeloma; and (g) distant metastases. Consider that some chronic diseases may affect blood markers, such as cirrhosis [14]. Of the 2,606 patients, 26 had cirrhosis, about 1%. Our study did not exclude these patients, because there is no uniform standard for the influence of chronic disease effects on blood markers. More detailed medical records are included in the Gastric Cancer Information Management System v1.2 of Harbin Medical University Cancer Hospital (Copyright No. 2013SR087424, http://www.sgihmu.com/).

We retrospectively analyzed the physical examination data of our hospital staff in 2018 as a healthy control group. None of the enrolled population had inflammatory diseases, history of antiplatelet agent therapy, and history of cancer and hematological diseases. In order to protect their privacy, we extracted data with the assistance of the medical examination center and the information center, without revealing personal information. This study was approved by the Ethics Committee of Harbin Medical University Cancer Hospital (2019-57-IIT). The need for informed consent was waived due to the retrospective nature of this study.

### 2.2. Hematology Analysis

Complete blood counting (CBC) with automated differential counts was performed for all patients and the healthy controls. On the day of admission or the morning of the second day, 2 ml of peripheral fasting blood was collected from the cubital vein, and the serum was separated for analysis. CBC test was performed within 4 hours. The NLR was calculated by dividing the absolute neutrophil count (ANC) by the absolute lymphocyte count (ALC); the PLR was calculated by dividing the absolute platelet (PLT) count by the ALC.

### 2.3. Statistical Analysis

Differences in clinical parameters by NLR, PLR, CEA, and CA19-9 were assessed by the chi-squared test. Continuous variables were expressed as mean ± standard deviation (SD). The diagnostic value of all biomarkers for GC was calculated and compared according to the receiver operating characteristic (ROC) curve, and the area under the curve (AUC) was also calculated. Binary logistic regression was used to combine inflammatory markers. *P* values less than 0.05 were considered statistically significant. Statistical analysis was performed using SPSS 22.0 for Windows (SPSS, Chicago, IL, USA).

## 3. Results

### 3.1. Clinicopathological Features of Patients with GC

Patient demographic, clinical, and pathological features are summarized in Table 1. Of the 2,606 patients, 1,811 (69.5%) were male and 795 (30.5%) were female. The numbers of patients with Borrmann types 0, I, II, III, IV, and V were 163 (6.3%), 117 (4.5%), 498 (19.1%), 1,100 (42.2%), 158 (6.1%), and 49 (1.9%), respectively. The TNM staging results were as follows: 346 patients (13.3%) were in stage IA, 167 (6.4%) in stage IB, 318 (12.2%) in stage IIA, 319 (12.2%) in stage IIB, 397 (15.2%) in stage IIIA, 224 (12.8%) in stage IIIB, 317 (12.2%) in stage IIIC, and 167 (6.4%) in stage IV.

### 3.2. Systemic Inflammatory Markers and Tumor Markers

Compared with the healthy control group, ANC, PLT, CEA, and CA19-9 in GC patients were significantly increased, and ALC was significantly offset. In addition, PLR and NLR were higher than those in healthy controls (Table 2). According to the TNM staging, we found that although NLR and PLR increased with tumor progression similar to CEA and CA19-9, they increased more significantly in the early stage of tumor (Table 3). The patients and the control group were divided into two subgroups according to gender, and it was found that the differences in NLR and PLR in GC patients of different genders were statistically significant compared with the control group (Tables 4 and 5).

### 3.3. ROC Analysis of CEA, CA19-9, NLR, and PLR

**Figure 1:**
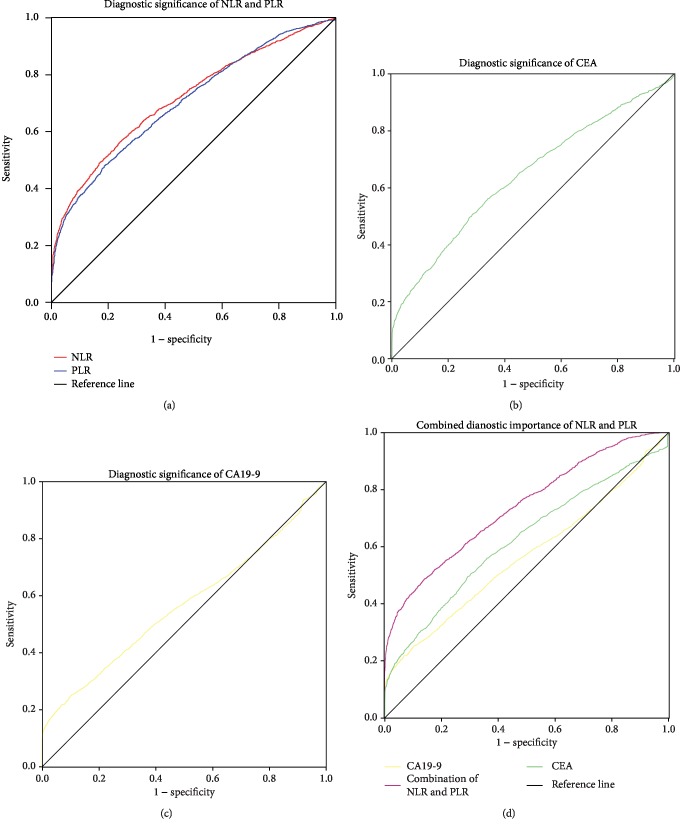
Comparison of systemic inflammatory markers and traditional tumor markers in the diagnosis of GC. NLR: neutrophil-lymphocyte ratio; PLR: platelet-lymphocyte ratio.

NLR and PLR showed diagnostic value in GC (Figure 1(a)), the cutoff value of the NLR for GC diagnosis was 2.258 (AUC = 0.715, 95% confidence interval (CI) = 0.703-0.728, Se = 48.88%, Sp = 83.04%), the cutoff value of the PLR was 147.368 (AUC = 0.707, 95%CI = 0.695‐0.719, Se = 48.20%, Sp = 81.79%), the cutoff value of CEA was 2.1 (AUC = 0.623, 95%CI = 0.609‐0.635, Se = 49.60%, Sp = 70.59%, Figure 1(b)), and the cutoff value of CA19-9 was 25.1 (AUC = 0.565, 95%CI = 0.552‐0.579, Se = 49.60%, Sp = 93.13%, Figure 1(c)). The AUC for combined detection with the NLR and PLR was 0.739 (95%CI = 0.727‐0.751, Se = 47.96%, Sp = 85.65%, Figure 1(d)). Using the Bonferroni correction, the NLR and PLR were superior to CEA and CA19-9 in the diagnosis of GC, either alone or in combination (*P* < 0.0001).

### 3.4. ROC Analysis of the NLR and PLR in Male and Female Patients

**Figure 2:**
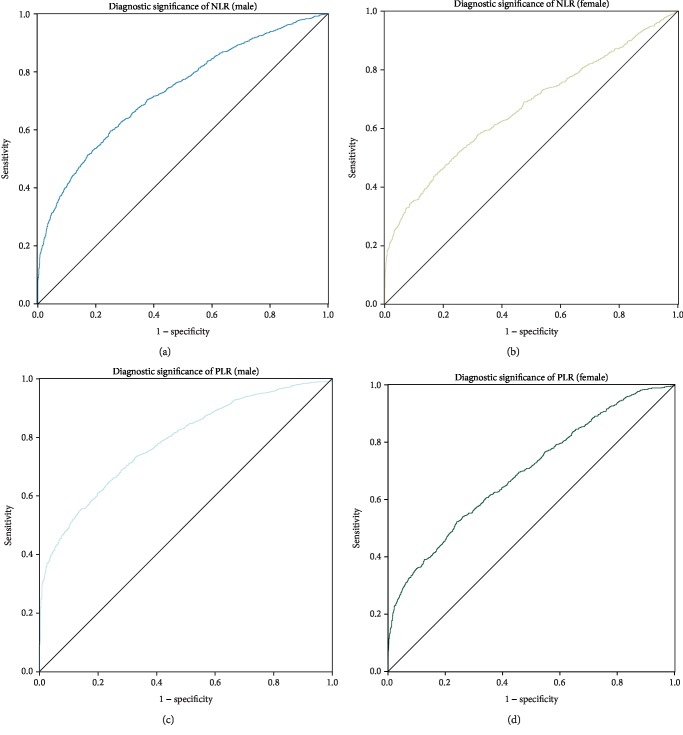
Patients and healthy controls were grouped by gender to calculate the value of NLR and PLR for the diagnosis of GC. NLR: neutrophil-lymphocyte ratio; PLR: platelet-lymphocyte ratio.

**Figure 3:**
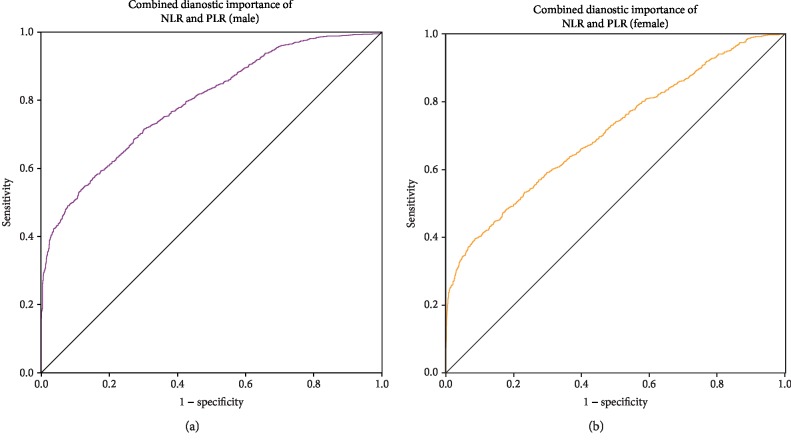
Patients and healthy controls were grouped by gender to calculate the value of combined NLR and PLR for the diagnosis of GC. NLR: neutrophil-lymphocyte ratio; PLR: platelet-lymphocyte ratio.

The cutoff value of the NLR for male GC patients was 2.069 (AUC = 0.732, 95%CI = 0.715‐0.749, Se = 59.49%, Sp = 74.81%, Figure 2(a)). The cutoff value for female patients with GC was 2.248 (AUC = 0.669, 95%CI = 0.649‐0.685, Se = 44.15%, Sp = 82.62%, Figure 2(b)). The cutoff value of the PLR for male GC patients was 133.333 (AUC = 0.783, 95%CI = 0.769‐0.801, Se = 55.57%, Sp = 85.84%, Figure 2(c)). The cutoff value for female GC patients was 147.517 (AUC = 0.693, 95%CI = 0.680‐0.715, Se = 52.56%, Sp = 76.68%, Figure 2(d)). The AUC of the NLR and PLR combined for the diagnosis of male GC patients was 0.793 (95%CI = 0.771‐0.802, Se = 52.57%, and Sp = 88.43%, Figure 3(a)). The AUC of NLR and PLR combined for the diagnosis of female GC patients was 0.710 (95%CI = 0.693‐0.728, Se = 40.08%, Sp = 91.18%, Figure 3(b)). The combination of the NLR and PLR is more effective in diagnosing male patients with GC.

## 4. Discussion

As most GC patients are asymptomatic until the disease progresses to advanced stages [15, 16], the early diagnosis is considered a core issue in some important medical associations, such as the UICC, AJCC, and the Japanese Gastric Cancer Association (JGCA) [17, 18]. At present, gastroscopy is still the most effective method for guideline recommendation, but due to the uncomfortable experience and high economic burden, a widespread application in screening for early cancer detection is difficult [19, 20]. The most frequently used tumor markers CEA and CA19-9 are not unique for GC diagnosis due to their poor sensitivity and specificity [21].

The systemic inflammatory response accompanies the development of cancer, whether early or advanced cancer, which provides us with new methods for early identification of GC [22–24]. Previous studies have supported the hypothesis of “inflammation-cancer transformation” [25–27]: prolonged gastritis may cause GC [28]; chronic viral hepatitis can result in cirrhosis, which in turn leads to liver cancer [29]. The gastrointestinal tract epithelium is continuously exposed to the external environment and is susceptible to inflammation due to various pathogens or other stimuli [30]. Pathologists often observe the phenomenon of “cancer-related inflammation” in paraffin sections of gastrointestinal cancer [22, 31]. In view of the simple evaluation of tumor cell progression by the traditional TNM system, in 2012, French scientist Galon [32] proposed the incorporation of TNM immune, which is the infiltration of immune cells in pathological sections, into traditional TNM staging to determine the sensitivity to chemotherapy in postoperative patients. However, due to differences in central testing techniques, heterogeneity of immune cell distribution, inconsistent paraffin section selection criteria, and nonobjective quantitative immune infiltration, proposing a widely accepted criterion for clinical applications is difficult [10]. Therefore, is it possible to acquire more accurate cancer information from blood samples by a relatively convenient detection method using uniform measurement criteria?

In 1869, Ashworth first proposed the concept of CTC. After years of research, CTC testing was first approved for clinical use in China in 2012. However, the rarity, heterogeneity and high cost of CTC testing pose challenges in using them as biomarkers [33–35]. This has led researchers to seriously consider the phenomenon that cancer cells enter the peripheral blood at the initial stage of cancer, but early metastasis is rare [36, 37]. Recent publications have reported that CTC entering peripheral blood triggers an immune response including an increase in the proportion of cancer-associated macrophages and neutrophils. Li [38] observed an increase in the proportion of peripheral blood neutrophils in the progression of malignant tumors, which was associated with prognosis. Burr [39] pointed out that nonsteroidal anti-inflammatory drugs can reduce the risk of systemic inflammation and tumorigenesis. This relationship between systemic inflammation and tumors has been of interest to researchers. The NLR and PLR, which are markers of systemic inflammation, are expected to aid the early diagnosis of GC.

Neutrophils constitute 50-70% of all white blood cells in the human circulation with an average lifespan of 5.4 days in the homeostatic condition of oxidative stress response [40]. They are currently believed to promote cancer cell proliferation, vascularization, and metastasis by producing proangiogenic chemokines and vascular endothelial growth factor [41, 42]. Lymphocytes in peripheral blood are currently thought to cause synergistic cytotoxicity and exert tumor suppressor properties [43]. In addition, platelets are known to be involved in tumor development [44]. Neonatal cancer cells promote platelet production and activation by secreting active substances such as interleukin-6, while activated platelets secrete vascular endothelial growth factor, platelet-derived growth factor, and transforming growth factor-*β* to promote cancer angiogenesis [45–47]. In this study, we found that the NLR was significantly higher in patients with GC than in normal subjects, which also indicated that the neutrophil-based protumor inflammatory response in the peripheral blood of tumor patients was stronger than the lymphocyte-based antitumor immune effect.

In the present study, it was found that the systemic inflammatory markers NLR and PLR were more valuable for the diagnosis of GC than the traditional tumor markers CEA and CA19-9. Similar researches indicated that the combination of preoperative NLR, PLR, and traditional tumor biomarkers could significantly improve the diagnostic efficacy not only for early stage colorectal cancer [48] but also for oral cavity cancer [49], ovarian cancer [50], and hepatocellular cancer [51]. Researchers found that the combination of NLR and PLR in the colorectal cancer patients with TNM stage I and II was higher than that in the healthy controls, and patients with stage III had a higher NLR and PLR than those with stage I and II. In nonmalignant diseases, such as predicting all-cause mortality of acute pulmonary embolism [52], assessing the inflammatory response of patients with systemic lupus erythematosus [53], and predicting the prognosis of patients with glomerulonephritis [54], both NLR and PLR have clinical applicability. Moreover, this is the first study in a Chinese population, and to our knowledge is the first to compare tumor markers with inflammatory markers. Further analysis revealed that the increase in NLR and PLR values was associated with higher TNM stage, which was similar to the findings by Li [55]. The systemic inflammatory markers were more markedly elevated in stage I of GC than conventional tumor markers. This indicates that the inflammatory markers in peripheral blood are expected to be an important diagnostic basis for early screening of GC.

We found that the systematic inflammatory markers NLR and PLR had higher diagnostic value in male GC patients than in female GC patients. This may be due to the immune system in males having a more intense “reaction” to cancer cells entering the peripheral blood, leading to an increase in the proportion of neutrophils and platelets. It is acceptable that the inclusion of gender factors in the early diagnosis of GC can supplement previous research and improve the application value of systemic inflammatory markers.

This study also had some limitations. GC is one of the most widely distributed cancers in different regions, which makes it difficult to draw a generalized conclusion in a single-center [56, 57]. At the same time, all patients in our study were Asians, and whether the results of this study can be generalized to white and black population needs further study. Furthermore, this study did not discuss the helicobacter pylori infection and did not include patients with erosive gastritis in the control group to evaluate whether the findings of this study would be affected by local inflammatory infection. However, many current studies on “noninflammatory tumors” such as breast cancer and thyroid cancer have demonstrated that the NLR and PLR are associated with tumor progression [58, 59]. This may indicate that cancer cells entering the peripheral blood can cause changes in NLR and PLR values, and local inflammation is not necessary for these changes. Although our research has a long way for supplementing international guidelines for the diagnosis and treatment of GC, it does not prevent the clinician from applying systemic inflammatory markers as a method for the screening and identification of high-risk populations. We suggest that further studies should not only focus solely on finding peripheral cancer cells or their “secretion” as a diagnostic tool but also include systemic inflammation caused by tumor cells in the diagnostic criteria for GC.

## 5. Conclusions

In summary, our results demonstrated that the NLR and PLR, markers of systemic inflammation, were associated with the diagnosis of GC, specifically in male patients. These findings suggest that systemic inflammatory markers in peripheral blood samples can benefit high-risk populations with GC. Existing diagnostic methods should be combined with the assessment of systemic inflammatory markers in clinical practice.

## Figures and Tables

**Table 1:** Characteristics of GC patients and healthy controls.

	Patients	Healthy controls	*P* value
Sex			<0.05
Male	1,811	1,087	
Female	795	22 14	
Age (years)			<0.05
	59.29 ± 9.86	46.16 ± 14.09	
Borrmann type^a^			
0	163		
I	117		
II	498		
III	1,100		
IV	158		
V	49		
AJCC stage^b^			
IA	346		
IB	167		
IIA	318		
IIB	319		
IIIA	397		
IIIB	334		
IIIC	317		
IV	167		

^a^Borrmann 0: progressive GC with superficial spreading tendency; Borrmann V: cannot be classified into other types of GC. ^b^AJCC: 8th edition of the Cancer Staging Manual of the American Joint Committee on Cancer.

**Table 2:** Laboratory values in GC patients and healthy controls.

	Patients	Healthy controls	*P* value
WBC	6.97 ± 2.32	6.45 ± 1.68	<0.05
ANC	5.51 ± 7.93	3.69 ± 1.26	<0.05
ALC	1.98 ± 2.17	2.21 ± 0.62	<0.05
PLT	273.27 ± 104.96	247.11 ± 58.68	<0.05
NLR	3.23 ± 4.14	1.74 ± 0.64	<0.05
PLR	167.11 ± 88.99	118.28 ± 36.79	<0.05
CA19-9	58.80 ± 174.57	11.61 ± 10.40	<0.05
CEA	13.66 ± 71.14	2.01 ± 8.33	<0.05

WBC: white blood cell; ANC: absolute neutrophil count; ALC: absolute lymphocyte count; PLT: blood platelets; NLR: neutrophil-lymphocyte ratio; PLR: platelet-lymphocyte ratio.

**Table 3:** Laboratory values of systemic inflammation in different TNM stages.

	Healthy controls	Stage I	Stage II	Stage III	Stage IV
NLR	1.74 ± 0.64	2.86 ± 4.48	3.14 ± 3.64	3.31 ± 3.99	3.66 ± 4.78
PLR	118.23 ± 36.74	135.38 ± 53.84	163.74 ± 82.31	174.14 ± 85.12	196.74 ± 122.65
CA19-9	11.61 ± 10.40	11.93 ± 15.09	37.26 ± 126.70	74.63 ± 190.33	183.71 ± 332.46
CEA	2.01 ± 8.33	2.69 ± 6.51	6.68 ± 37.98	17.63 ± 78.91	41.49 ± 127.56

**Table 4:** Laboratory values in male GC patients and healthy controls.

	Patients	Healthy controls	*P* value
Number	1811	1087	
WBC	7.13 ± 2.27	6.78 ± 1.76	<0.05
ANC	5.69 ± 8.15	3.84 ± 1.30	<0.05
ALC	2.03 ± 2.36	2.30 ± 0.71	<0.05
PLT	269.68 ± 112.32	223.80 ± 51.51	<0.05
NLR	3.26 ± 3.80	1.77 ± 0.67	<0.05
PLR	164.61 ± 91.33	103.03 ± 30.10	<0.05

WBC: white blood cell; ANC: absolute neutrophil count; ALC: absolute lymphocyte count; PLT: blood platelets; NLR: neutrophil-lymphocyte ratio; PLR: platelet-lymphocyte ratio.

**Table 5:** Laboratory values in female GC patients and healthy controls.

	Patients	Healthy controls	*P* value
Number	795	2214	
WBC	6.63 ± 2.40	6.29 ± 1.62	<0.05
ANC	5.10.±7.39	3.61 ± 1.23	<0.05
ALC	1.89.±1.62	2.16 ± 0.57	<0.05
PLT	281.44.±85.28	258.56 ± 58.60	<0.05
NLR	3.15.±4.84	1.73 ± 0.62	<0.05
PLR	172.80 ± 83.11	125.77.±37.45	<0.05

WBC: white blood cell; ANC: absolute neutrophil count; ALC: absolute lymphocyte count; PLT: blood platelets; NLR: neutrophil-lymphocyte ratio; PLR: platelet-lymphocyte ratio.

## Data Availability

The datasets used in this study are available from the corresponding author on reasonable request. More information can also be obtained from the Gastric Cancer Information Management System v1.2 of Harbin Medical University Cancer Hospital (Copyright No. 2013SR087424, http://www.sgihmu.com/).
